# A systematic review of kidney-on-a-chip-based models to study human renal (patho-)physiology

**DOI:** 10.1242/dmm.050113

**Published:** 2023-06-19

**Authors:** Vivian V. T. Nguyen, Vasiliki Gkouzioti, Christian Maass, Marianne C. Verhaar, Robin W. M. Vernooij, Bas W. M. van Balkom

**Affiliations:** ^1^ Department of Nephrology and Hypertension, UMC Utrecht, 3584CX Utrecht, The Netherlands; ^2^ esqLABS GmbH, 26683 Saterland, Germany; ^3^ Julius Center for Health Sciences and Primary Care, UMC Utrecht, Utrecht University, 3584CX Utrecht, The Netherlands

**Keywords:** Microphysiological models, Nephron segments, Toxicology, *In vitro* disease modeling

## Abstract

As kidney diseases affect ∼10% of the world population, understanding the underlying mechanisms and developing therapeutic interventions are of high importance. Although animal models have enhanced knowledge of disease mechanisms, human (patho-)physiology may not be adequately represented in animals. Developments in microfluidics and renal cell biology have enabled the development of dynamic models to study renal (patho-)physiology *in vitro*. Allowing inclusion of human cells and combining different organ models, such as kidney-on-a-chip (KoC) models, enable the refinement and reduction of animal experiments. We systematically reviewed the methodological quality, applicability and effectiveness of kidney-based (multi-)organ-on-a-chip models, and describe the state-of-the-art, strengths and limitations, and opportunities regarding basic research and implementation of these models. We conclude that KoC models have evolved to complex models capable of mimicking systemic (patho-)physiological processes. Commercial chips and human induced pluripotent stem cells and organoids are important for KoC models to study disease mechanisms and assess drug effects, even in a personalized manner. This contributes to the Reduction, Refinement and Replacement of animal models for kidney research. A lack of reporting of intra- and inter-laboratory reproducibility and translational capacity currently hampers implementation of these models.

## Teaser

**Summary:** Kidney-on-a-chip-based *in vitro* models have greatly evolved and provide valuable instruments to study human kidney (patho-)physiology, contributing to the Reduction, Replacement and Refinement of animal experiments.

## INTRODUCTION

Kidney disease impacts the worldwide population in pandemic-like proportions, with an estimated one in ten individuals having chronic kidney disease (CKD), often without realizing it (Luyckx et al., 2018). CKD is mainly caused by diabetes mellitus, hypertension, and drug- or ischemia–reperfusion-induced acute kidney injury, and genetic diseases, like polycystic kidney disease (Lv and Zhang, 2019; Neyra and Chawla, 2021; Pepper and Trompeter, 2022). CKD is associated with high mortality and severely impacts quality of life. Its progressive nature is thus far irreversible, and patients reaching end-stage kidney disease need kidney replacement therapy and can only be cured by receiving a donor kidney via transplantation. Laboratory models to study potential causes of CKD, drug efficacy and toxicity, and disease etiology and progression, and to develop (regenerative) therapies or dialysis modalities, are essential. CKD can be considered a systemic disease, involving organs other than the kidneys, and many CKD patients suffer from cardiovascular complications (Schiffrin et al., 2007). To adequately model the systemic effects in CKD, many different animal models to study inherited kidney diseases and CKD have been developed, including zebrafish, rodent, goat and porcine models (van Koppen et al., 2013; Korstanje et al., 2014; Chade et al., 2021; Oliveira et al., 2021; Hopp et al., 2022).

### *In vitro* models

For studies into the molecular and cell biological aspects of kidney diseases, *in vitro* cell models are indispensable, next to observations from animal experiments. The broad availability and relative ease of handling make cell models valuable tools to study general cell physiological aspects and specific consequences of mutations occurring in genetic diseases. Many cell lines generated from animal or human kidney cells are available, each representing a specific nephron segment, like glomerular MPC5 podocytes (Mundel et al., 1997), conditionally immortalized proximal tubule cells (Wilmer et al., 2010) and Madine-Darby canine kidney (MDCK) collecting duct cells (Gaush et al., 1966).

Although they are very useful for elucidating general cell biological and biomolecular mechanisms, cell lines often lose some of the original cell characteristics and do not allow the study of patient heterogeneity. Many researchers therefore opt to use primary cells, which are directly obtained from donor tissue or can be obtained commercially. Although such cells, like renal proximal tubule epithelial cells, more closely resemble *in vivo* physiology and patient heterogeneity, they are limited in proliferation capacity and overall lifespan. Recent developments in induced pluripotent stem cell (iPSC) and organoid biology may, however, offer cell types that combine patient specificity with a long lifespan (Takahashi and Yamanaka, 2006; Takahashi et al., 2007; Sato et al., 2009; Freedman et al., 2013; Lancaster and Knoblich, 2014; Freedman, 2015; Mae and Osafune, 2015; Takasato et al., 2015).

### Kidney-on-a-chip

The use of dynamic culture conditions to resemble renal cells' physiological conditions more closely has provided detailed insights into kidney cell biological mechanisms in health and disease. These kidney-on-a-chip (KoC) models have been used to study physiological parameters ranging from flow and shear stress to organ–organ interactions in multi-organ-on-a-chip (MOC) models (Ingber, 2022). With the increased understanding that animal models do not always reflect human physiology (Mak et al., 2014; Powell, 2018), the use and development of MOC models is gaining more and more interest. As a result, a wide variety of kidney-based MOC models have been developed (Irvine et al., 2021).

Despite the increased interest in, and availability of, kidney-based organ-on-a-chip (OoC) models, evidenced by the growing number of publications, no systematic overview describing the evolution and potential of such models is available. Here, we provide a comprehensive overview of the evolution and current state-of-the-art of *in vitro* KoC models, ranging from basic flow models to elucidate cell biological mechanisms to complex MOC models, and their use to mimic human (patho-)physiology and pharmacological effects.

## RESULTS

### Literature search and selection

**Fig. 1.**
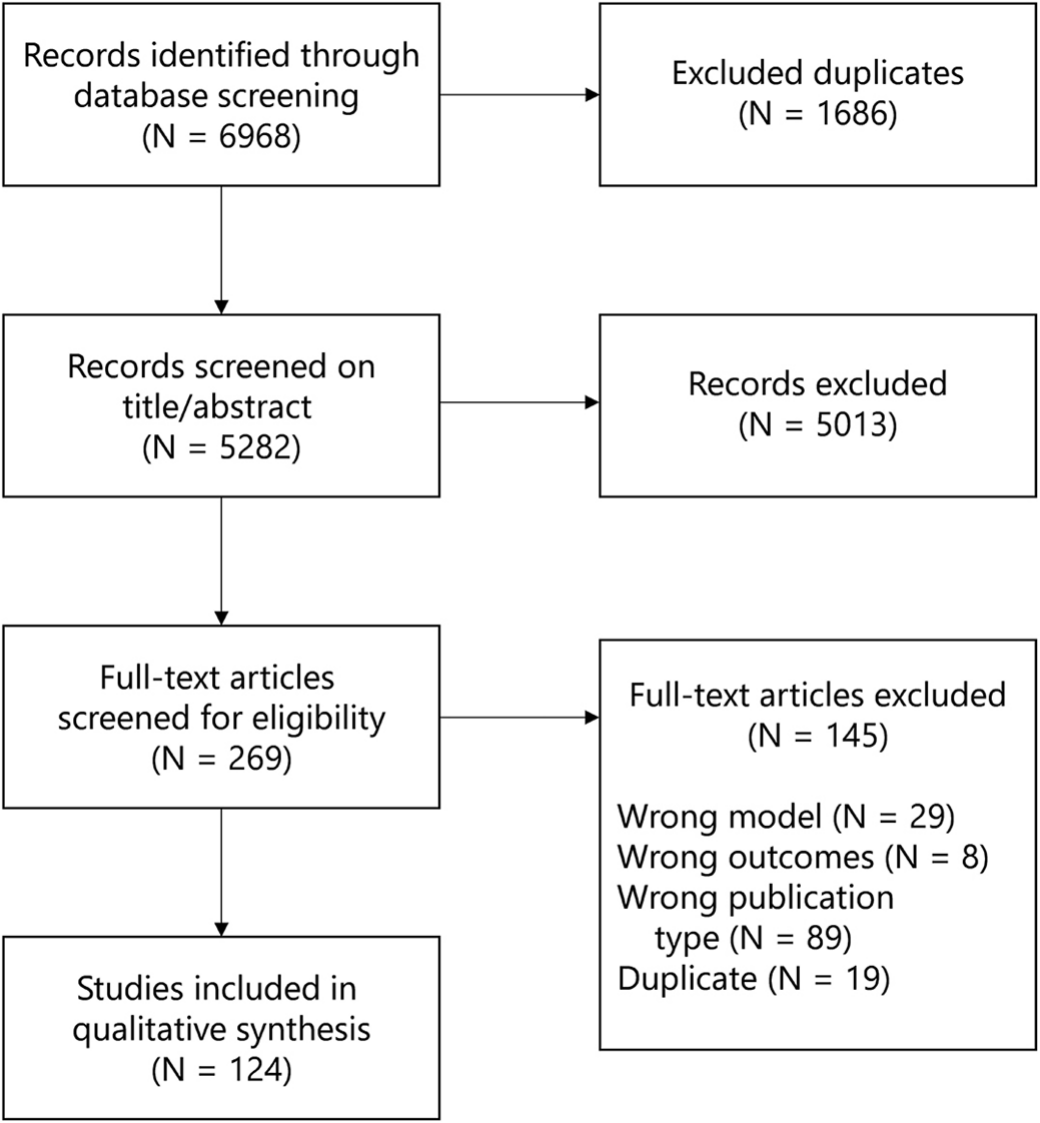
**PRISMA study selection flow diagram.** Search and selection strategy for this study, resulting in the selection of 124 studies. PRISMA, Preferred Reporting Items for Systematic Reviews and Meta-Analyses.

As depicted in Fig. 1, 6968 publications were identified in two databases. From these, 1686 duplicates were excluded, and 5282 records were screened based on title and abstract, resulting in the exclusion of 5013 records not meeting predefined eligibility criteria. Subsequently, 269 full-text articles were screened, of which 145 were excluded for reasons as described (Fig. 1; Table S1). In total, 124 studies were included for qualitative synthesis (Table S2).

### Narrative synthesis

#### Represented nephron segments

**Fig. 2.**
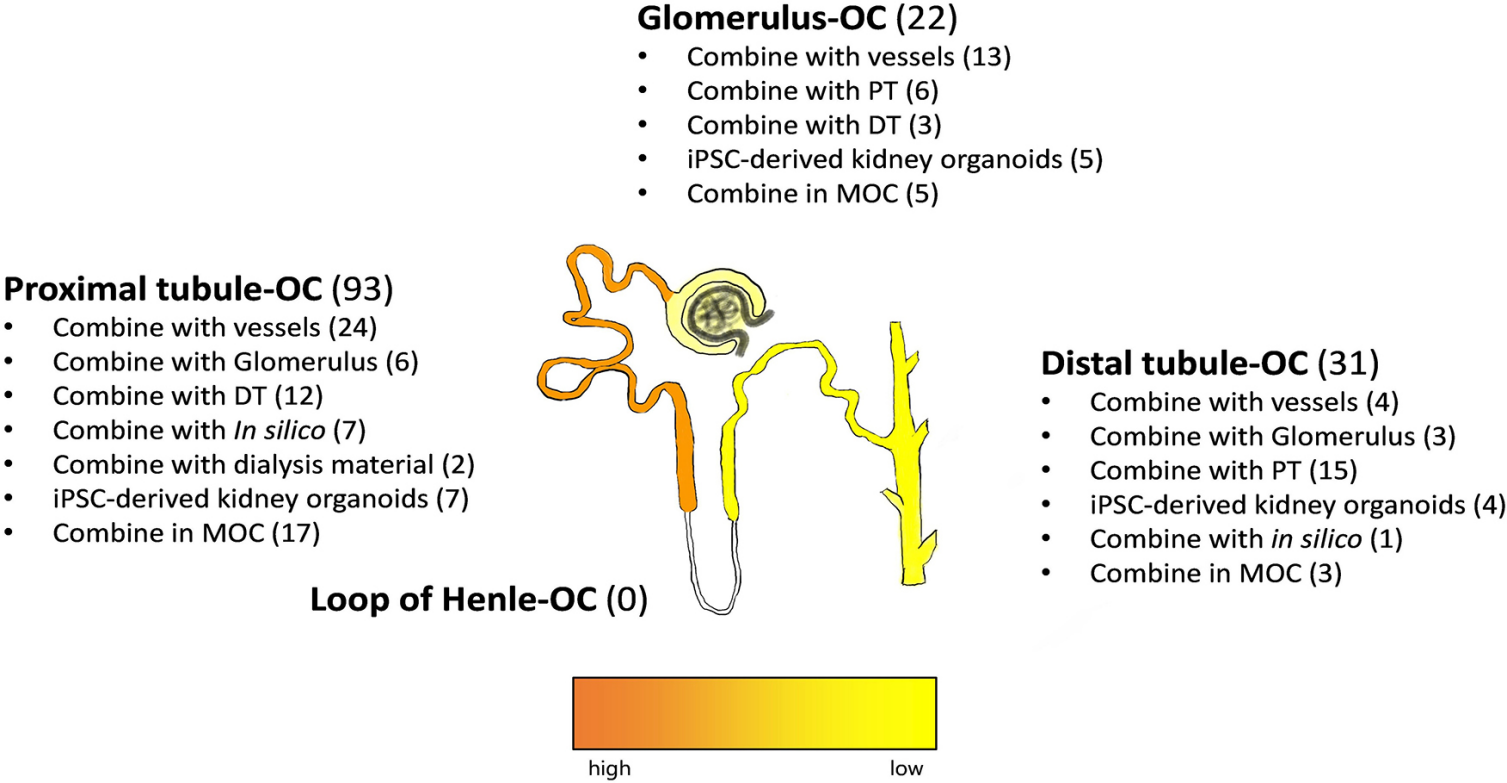
**Representation of nephron segments in kidney-on-a-chip models.** Most kidney-on-a-chip (KoC) models are based on proximal tubule (PT) cells, followed by models representing the distal segments and the glomerulus. This trend is observed for single organ-on-a-chip (OoC) and for multi-organ-on-a-chip (MOC) models. DT, distal tubule; iPSC, induced pluripotent stem cell; OC, on-a-chip.

The nephron, including the glomerulus, Bowman's capsule, proximal convoluted tubules, loop of Henle and distal tubules, constitutes the kidney's basic functional unit. Establishing a comprehensive *in vitro* model of its complex architecture, including spatial distribution of several cell types with their specific functions, represents a huge challenge. Indeed, none of the 124 included manuscripts described the establishment of an *in vitro* model for the entire nephron. Researchers rather focused on a nephron segment of relevance, depending on the research question (Fig. 2). For instance, some groups studied glomerular maturation and filtration, while others investigated reabsorption, toxicity or disease effects in tubule segments. The nephron segment of highest interest is the proximal tubule (PT), which is a component in 92 publications. The glomerulus, mostly represented by podocytes, either or not in combination with endothelial cells, is described in 22 articles, whereas distal tubule segments, including the collecting duct, were mimicked in 31 included publications. It should be noted that the loop of Henle was represented in none of the included studies.

### Organ combinations

**Fig. 3.**
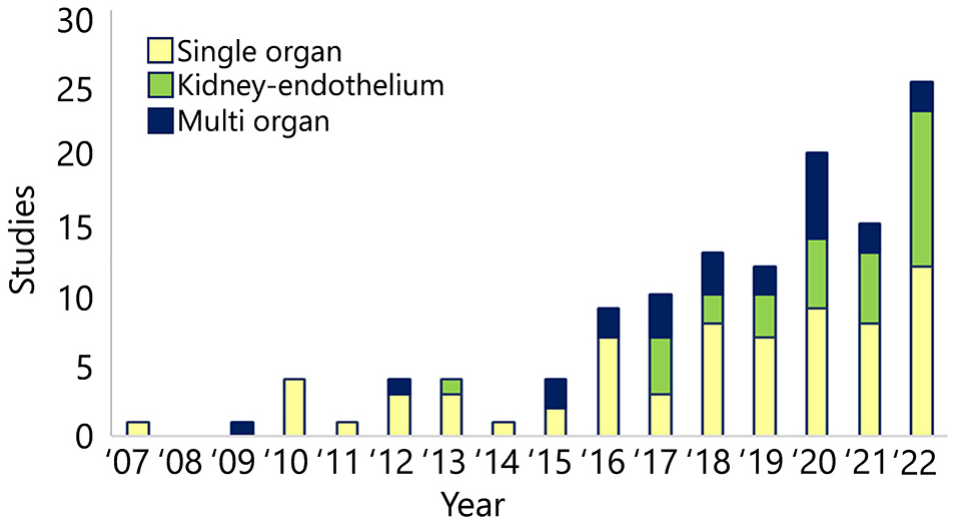
**Single and combined kidney-based OoC models.** Yearly publications describing microphysiological models including only renal cells (Single organ), and combinations with vasculature (Kidney-endothelium) or other organ (Multi organ) models.

The kidneys play a crucial role in regulating systemic homeostasis, and combined OoC models including a kidney representative allow *in vitro* mimicking of more complex processes such as drug metabolism and secretion (Ma et al., 2021). After the first KoC publication in 2007 (Baudoin et al., 2007), KoC publications remained limited until 2015, not exceeding four papers annually (Fig. 3). In 2016, the amount of publications had more than doubled and kept increasing throughout the years, with the highest being 25 published papers in 2022. Although single-organ KoC models remain most common (69/124 publications), since 2017, more than 38% of the yearly publications report the integration of other tissues/organs into OoC models, underscoring the interest towards more complex and physiologically relevant kidney-based models (Fig. 3). Although integration of renal vasculature may not be considered as a MOC setup, the combination of either the proximal tubule or glomerulus with endothelium is reported in 31 publications (Table S2). The addition of vasculature allows for a more physiological nephron construction while mimicking the renal interstitial micro-environment (Zhou et al., 2016; Chapron et al., 2020; Liu et al., 2022), allowing assessment of tubular drug secretion (Chapron et al., 2020; Imaoka et al., 2021) and modeling diseases involving renal peritubular microvessels (Liu et al., 2022).

Most reports on MOC models describe the combination of kidney with liver (21/24 papers), followed by publications describing the integration of intestine (9/24 papers) (Table S2). In 2009, the first report on a MOC system combined four organs (kidney, liver, lung and adipose tissue) in a multi-channel microfluidic system (Zhang et al., 2009)*.* In this model, kidney, liver and adipose tissue compartments had separate microfluidic channels, all eventually merging into one main channel leading to the lung compartment. The most popular organ combination, kidney and liver, is mostly used to study drug metabolism, excretion and toxicity, as recently highlighted by Irvine et al. (2021).

### Human KoC models and cell sources

**Fig. 4.**
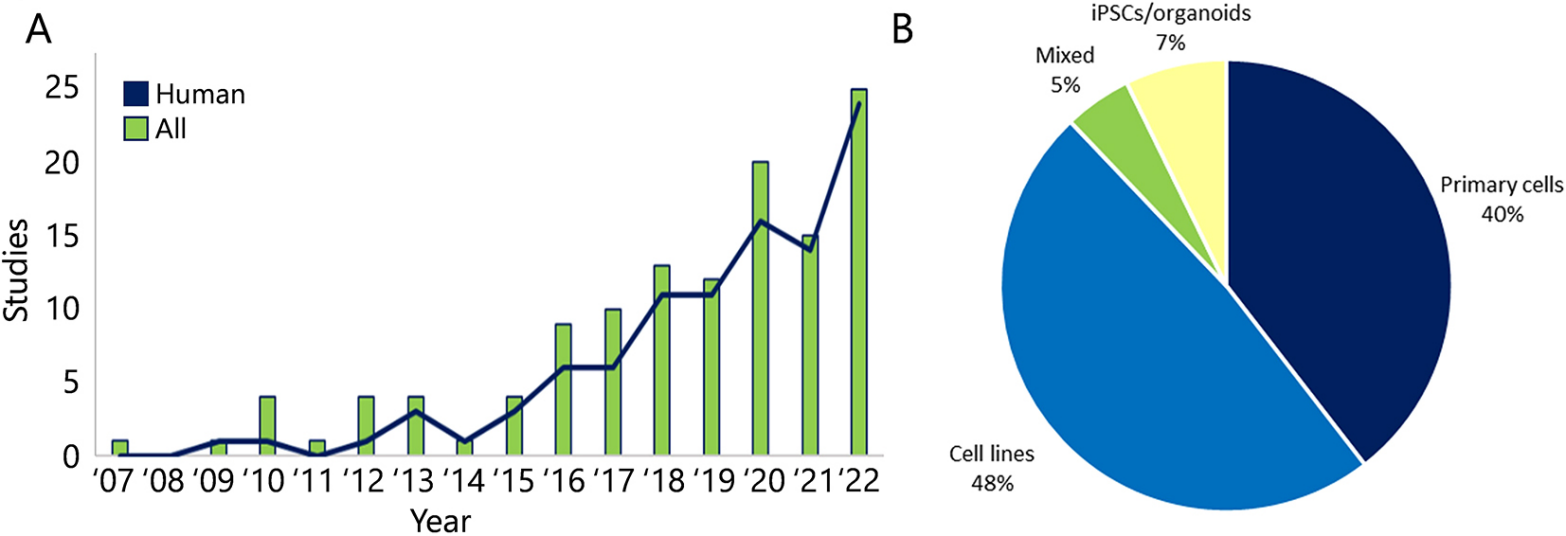
**Human KoC models and cell sources.** (A) Over time, the increased number of publications of KoC models has been especially driven by human-based rather than animal-based models. (B) Cell lines are the most common cell type used for KoC models, followed by primary cells, iPSC- or organoid-derived cells, and combinations of these.

Microphysiological systems, including OoC models, provide the potential to study cell biological and physiological mechanisms in human organ models, and help in the reduction and refinement of animal experiments. Along the reporting period, a transition from animal- to human-derived cells used in OoC models can be appreciated (Fig. 4A). The first KoC model was reported in 2007, describing a distal tubule dynamic model for toxicity testing based on MDCK cells (Baudoin et al., 2007). In 2009, the first report using human renal cells integrated in a microfluidic set up using the human-kidney-2 (HK-2) proximal tubular cell line to construct the renal compartment in an MOC model for drug screening (Zhang et al., 2009). After 2013, more than 55% of the annually published papers described human kidney cells to establish the renal compartment of OoC models. A similar evolution can be observed regarding the use of established cell lines, primary cells, and stem cell-derived cells and organoids. Cell lines have been widely used (60/124 papers) to develop KoC models, but clearly the use of primary cells (49/124 papers), and iPSC- and organoid-derived kidney cells (9/124 papers) has gained interest over time, with some publications describing the use of combinations of cell lines and primary cells (6/124 papers; Fig. 4B).

### Chip platforms

**Fig. 5.**
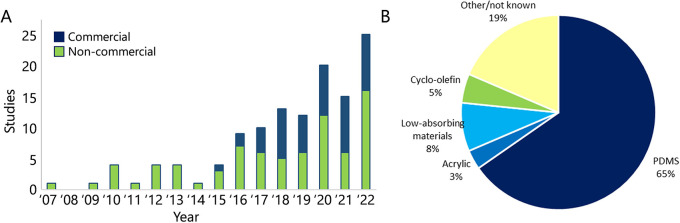
**Chip platforms.** (A) After an initial period of in-house-developed OoC platforms, since 2015, an increasing amount of publications describe the use of commercial platforms. (B) Evaluation of used chip materials indicates that polydimethylsiloxane (PDMS) is the most commonly used material for OoC platforms.

Initial KoC publications used in-house-made microfluidic devices, until the first report employing a commercially available microfluidic chip was published in 2015 (Maschmeyer et al., 2015) (Fig. 5A)*.* In this study, a microfluidic platform from TissUse GmbH was used to establish a four-organ (intestine, liver, skin and kidney) MOC model. The availability and use of commercially available microfluidic systems has increased since 2016, with currently around half of the publications utilizing such systems. Regardless of the origin of microfluidic platforms, the majority (81/124 papers) were fabricated using polydimethylsiloxane (PDMS) (Fig. 5B). In 2013, the first non-PDMS-based renal dynamic model, using a multi-layer biochip using Ti plates, containing HK-2 cells and human umbilical vein endothelial cells aiming at hemodialysis applications, was developed (Zhu et al., 2013). Other publications using a non-PDMS-based microfluidic platform based their fabrication on low-absorbing materials (Suter-Dick et al., 2018; Vormann et al., 2018; 2021; Vriend et al., 2018; 2020; Petrosyan et al., 2019; Schutgens et al., 2019; Gijzen et al., 2021; Naik et al., 2021) (10/124 papers), cyclo-olefin (Theobald et al., 2018, 2019; Yeste et al., 2018; Azizgolshani et al., 2021) (6/124 papers) or acrylic (Miller and Shuler, 2016; Chen et al., 2018; Ishahak et al., 2020) (4/124 papers).

### Functional assays and disease modelling

The main purpose of most included studies was to demonstrate that a certain material, chip design or cell source can be employed in an OoC model to study functional or developmental aspects of kidney biology, while others studied drug- or stress-induced renal injury or toxicity (53/124 publications) or human diseases (14/124 publications). Without exception, functional parameters were used to demonstrate effects on kidney physiology, ranging from cellular organization and polarization to viability and/or injury and therapeutic drug effects (Table 1; Table S2).

**Table 1.**
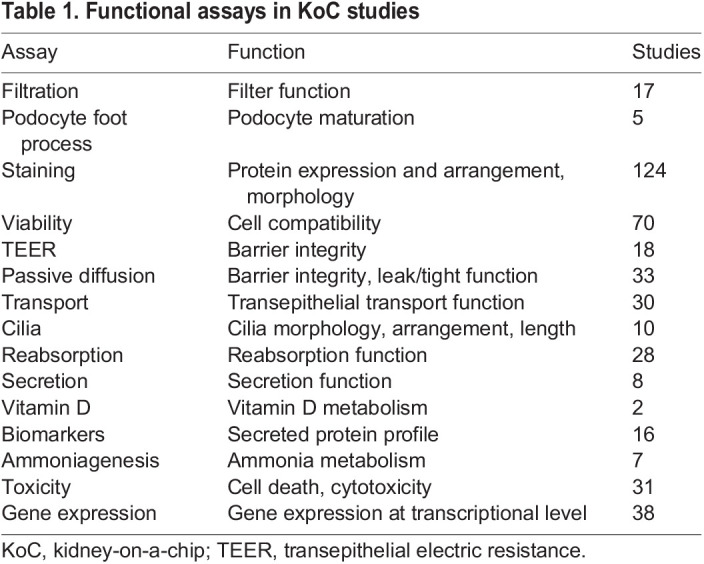
Functional assays in KoC studies

KoC models focusing on the glomerulus mainly use glomerular filtration barrier as a functional readout. Most researchers (16/22 of glomerulus KoC models) employed filtration assays, with albumin the most filtered substance. Only four studies verified podocyte maturation by analyses of foot processes by electron or fluorescence microscopy.

Functional assessment of renal tubular segments, represented in most studies, mostly consists of evaluating the selective barrier established by this epithelium (Qi et al., 2007). Passive diffusion assays using substrates such as fluorescein isothiocyanate (FITC) coupled to inulin or dextran were used in 33 and 24 studies describing proximal and distal tubule KoC models, respectively. Assessment of epithelial barrier function by transepithelial electric resistance (TEER) was employed in 13 studies. The selective permeability, one of the most important characteristics of the renal tubule, established by the expression of specific transporter proteins, was assessed in only 25 articles describing proximal tubule-based KoC models. These include assays to assess the functional transport of several substrates like fluorescent glucose analogs (for SGLT2; also known as SLC5A2), 5-chloromethylfluorescein diacetate (CMFDA) or 5(6)-carboxy-2′,7′-dichlorofluorescein (CDFDA) for MRP2 (also known as ABCC2) and MRP4 (also known as ABCC4), or calcein-AM (for PGP). Basic renal functions, such as ammoniagenesis (seven studies) and vitamin D metabolism (two studies), were also evaluated. In 24 MOC studies, assays to assess the function of other organs than the kidney were performed. TEER measurements (four studies), passive diffusion assays (four studies) and active transport assays [for MRP2/4, PGP and OAT1/3 (also known as SLC22A6/8)] (two studies) were used to evaluate the function for intestinal epithelium. Also, liver albumin secretion (five publications) and cellular metabolism (ten publications) were employed intensively. It is important to note that the expression of targeted molecules was intensively focused on at protein expression level, using staining methods, in every study. In addition, 38 studies looked at that at transcriptional level, either by quantitative PCR (27 studies), RNA sequencing (ten studies) or whole-genome sequencing (one study).

### Quality assessment

**Fig. 6.**
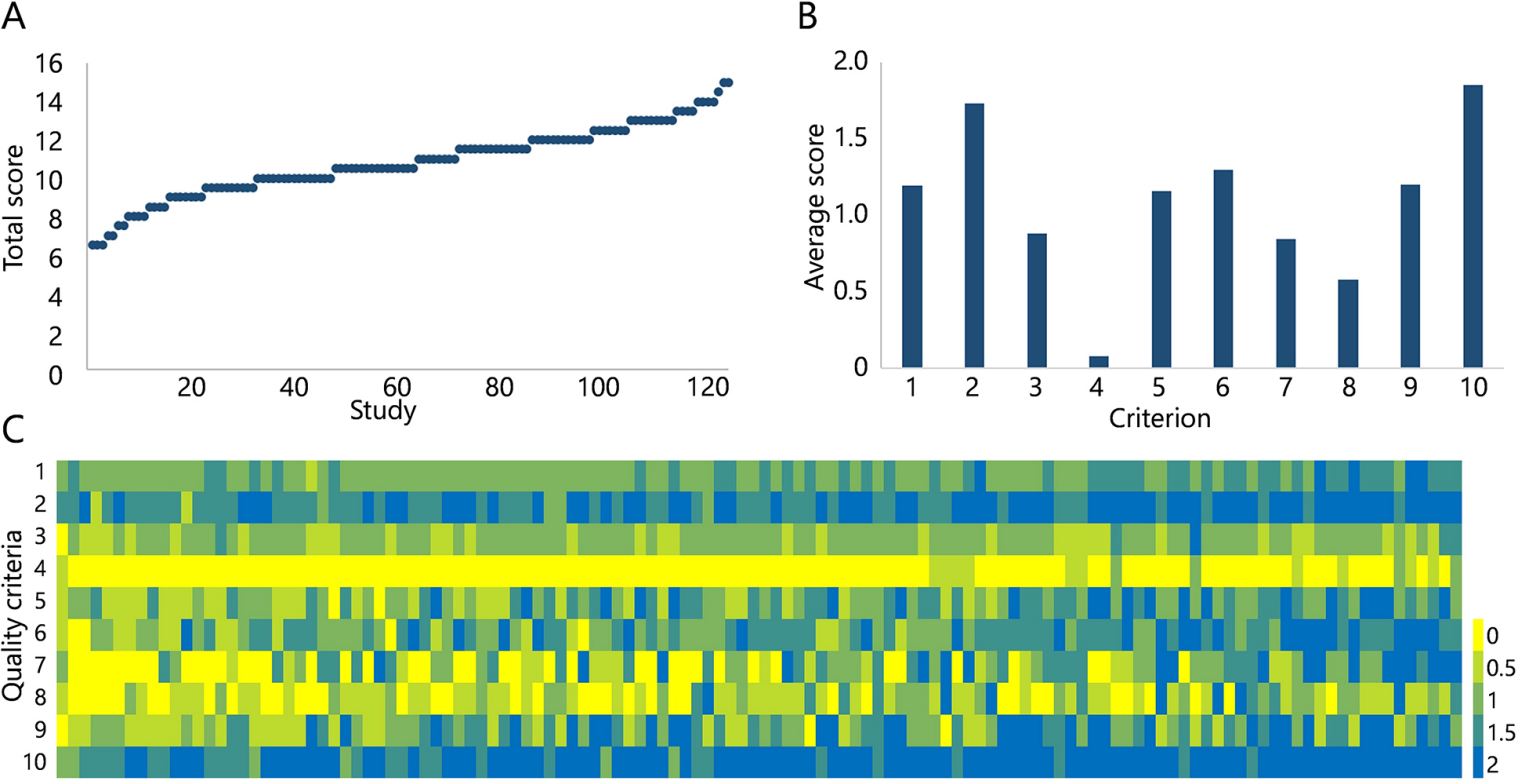
**Quality scoring.** (A,B) Total quality score distribution of all included studies (A) and average scores for each criterion (B). (C) Heatmap displaying the quality scores for each item (vertical) of each publication, ranked from high (left) to low (right).

We assessed the quality of the included studies using an adapted version of the ‘validation score’ as described (Irvine et al., 2021) (Table S3). The score, based on ten criteria, provides an indication of the quality of reporting and reproducibility (Fig. 6; Table S4). Total quality scores varied between 6.5 and 15 points, and the overall average was 10.8±1.8 points. The highest average scores were observed for criterion 2 concerning the clear description of protocols, and criterion 10 regarding the relevance of the test method (1.73±0.32 points and 1.85±0.28 points, respectively). The lowest scoring criterion concerned attempts to reduce investigator bias (criterion 4; 0.8±0.22 points), while criteria 3, 7 and 8, concerning reproducibility, disease and intervention modeling, and *in vitro–in vivo* extrapolation scored less than 1 point (i.e. 0.88±0.28 points, 0.85±0.74 points and 0.58±0.55 points, respectively).

## DISCUSSION

Here, we provide a comprehensive overview of literature describing OoC models mimicking renal (patho-)physiology. Kidney physiology in health and disease has been studied using *in vivo* animal models, *in vitro* cell models and human clinical studies. Its complex architecture and function comprise major challenges to comprehensively study kidney (patho-)physiology using *in vitro* models. Advances in microfluidics, 3D cell culturing, and stem cell and organoid biology are attracting a lot of scientific attention due to their potential for the development of complex, dynamic models more closely resembling specific aspects of renal biology.

### Representation of nephron segments

Focusing on specific research questions, ranging from basic cell biological aspects like polarization and development, to more applied aspects, such as drug metabolism, excretion and toxicity, different nephron segments have been modeled in OoC systems.

### Tubular segments

Most KoC models focus on the proximal tubule, the main site of active transport and reabsorption of solutes from urine coming from glomerular filtration. Packed with mitochondria and dependent on oxidative phosphorylation, this segment is particularly vulnerable to ischemic injury and mitochondrial toxicity. In contrast, more distal cells display higher Na^+^-K^+^-ATPase expression, crucial for ion and water reabsorption. Overall, OoC models representing the proximal tubule are mostly employed for drug efficacy and toxicity testing, for example for cisplatin (Jang et al., 2013) and antisense oligonucleotides (Nieskens et al., 2021), whereas OoC models mimicking distal segments are typically used to study basic cell biological aspects, such as the effects of shear stress on aquaporin-2 localization (Jang et al., 2011) or cyst formation (Verschuren et al., 2020).

### Glomerulus

Owing to challenges regarding the complexity of its structure and in sourcing the required cell types, the first glomerulus OoC model was reported in 2016 (Zhou et al., 2016), almost a decade later than models for tubule segments. To functionally mimic the glomerular filtration barrier, an orchestrated interplay between podocytes and endothelial cells has to be established, a combination observed in 15 of 22 OoC models mimicking the glomerulus. In contrast to OoC models employing tubular segments, glomerulus models are mostly used to study glomerular filtration in specific disease conditions, like diabetic or hypertensive nephropathy (Wang et al., 2017; Zhang and Mahler, 2021).

### Kidney-endothelium and MOC models

Other nephron segments also have a functional interaction with the renal vasculature, forming a complex structure with intricate microvascular capillary networks. Indeed, there are numerous reports describing OoC models combining nephron segments with vasculature: 26 of 93 proximal tubule OoC models, and three of 29 OoC models representing distal segments, integrated endothelial cells.

Besides the integration of vasculature, combining kidney models with other organ models enhances the complexity and physiological relevance of OoC models. Strikingly, the first report of an MOC model including the kidney was published in 2009 (Zhang et al., 2009). In this report, cell lines representing four human organs (kidney, liver, lung, fat) were functionally combined, providing proof of principle that complex *in vitro* models to investigate human physiology and assess food and drug safety are viable. After 2015, coinciding with the increased use of commercial chip platforms, a steady incline in the number of publications reporting kidney-based MOC models is observed. Intriguingly, the vast majority of these studies employed human cell models, strongly indicating the eagerness to develop *in vitro*, human measurement models to substitute animal testing, mostly focusing on combining kidney and liver, and employed to study pharmacokinetics, pharmacodynamics and toxicity, such as cisplatin pharmacokinetics (Herland et al., 2020) and chloroacetaldehyde toxicity (Leclerc et al., 2016).

### Renal cell sources

Advances in organoid and (induced)stem cell biology have greatly contributed to the development of human OoC models (van Berlo et al., 2021). Established human and animal cell lines like HK-2 and MDCK cells, with several publications employing primary cells, were mainly employed until 2017, after which an increased use of primary cells, and organoid- and iPSC-derived cells was observed. This trend further illustrates the potential of OoC models to mimic (patho-)physiological mechanisms in a human setting, which can even be employed in personalized medicine by using patient-derived (stem) cells and organoids (Schutgens et al., 2021; Zhou et al., 2021).

### Chip development

The first publication of a KoC model described the fabrication of a distal tubule-on-a-chip using MDCK cells cultured under flow conditions in a single-layer microchip generated by deep-reacting ion etching (Baudoin et al., 2007). Until 2015, all publications employed in-house fabricated (non-commercial) chip platforms for their KoC models, mostly using soft lithography or photolithography. PDMS was most commonly employed due to its ease of handling, transparency, biocompatibility and low-cost production (Halldorsson et al., 2015). However, PDMS can interact with hydrophobic compounds, potentially absorbing proteins and/or small molecules, like drugs, imposing a bias on the final experiment outcomes. Moreover, PDMS-based chips are hard to clean and sterilize after usage, thus they cannot be reused, rendering the cost of single experiments higher (Halldorsson et al., 2015). Nevertheless, although microphysiological systems have become more advanced over the past few years, also because of the application of 3D-printing technology, it is interesting that PDMS remains the dominant material for fabrication.

The use of commercialized chips can be advantageous regarding time and effort expenditure for academic research groups. There is a clear correlation between the increase in the number of KoC publications and the introduction of commercial chip platforms in 2015. Indeed, the availability of commercial platforms appears to indicate that an additional population of researchers could since then contribute, shifting the field from a technology-oriented field to a more biology-oriented playground. It should be noted that commercially available platforms are expensive and often provide a non-flexible design, limiting integration of either more biological compartments in a physiologically relevant setup, or accessories, like oxygen sensors or electrodes for barrier integrity measurements (Zhang et al., 2017). Despite the broad availability of commercial platforms, allowing identical setups in different laboratories, details on the hardware fabrication process of such platforms are regularly not provided.

### Functional assays

Representing a relevant parameter or function of modeled organs *in vitro* is essential for microphysiological systems, including KoC models. Indeed, most publications provide different levels of evidence that the presented model meets this criterion, with visualization, either with or without specific labeling, as the most common method. In addition, depending on the research question, functional characteristics directly related to represented nephron segments are assessed, including barrier function and specific transport (for tubule segments), and filtration and podocyte foot process analyses (for glomerulus models). In line with the complexity of KoC models, functional characterization ranges from the description of monolayer formation and cell maturation in earlier studies, to in-depth characterization of morphology, and barrier and transport function, sometimes even combined with an injury or disease model. It is important to note that in almost every study in which injury was induced, viability assays were performed, while levels of KIM-1 (also known as HAVCR1), a well-known marker protein for renal injury, were assessed in only five studies (Table S2). Although analyses of cell viability and renal injury may not directly relate to a specific renal function, they are essential when OoC models are employed to aid in the drug development process. Improving efficacy or screening for adverse events (toxicology) may be represented more accurately in human OoC models than in animal models, although clinical translation remains a challenge. Besides *in vitro* functional assays, six included studies employed computational, in combination with biological, models to bridge this translational gap. In one study (Maass et al., 2019), the authors developed an *in silico* virtual human twin model to describe cisplatin-induced kidney. *In silico* modeling was used to determine drug concentrations to be tested in the KoC model to recapitulate human-relevant response *in vitro*. Next, cisplatin-induced damage was modeled in the KoC platform, and viability and KIM-1 were assessed. The virtual twin approach was then employed to link the toxicodynamic effect to simulated drug concentrations and linked to clinical reports on elevated KIM-1 levels. This approach highlights the power of integrating human *in vitro* and *in silico* models, which can help to identify drug-related toxicity in preclinical studies, without actually testing the compound in humans.

### Quality/risk of bias

Owing to the heterogeneity of the included studies regarding used cell types, represented nephron segments, microfluidic platforms and functional analyses, meta-analyses could not be performed. Our adapted quality scoring questions, however, provided insight into the strengths and weaknesses of individual studies, and on quality aspects in general. It is clear that, in general, the study approach very well aligns with the aim of the study and the description of used protocols (criterion 10 and 2, respectively). However, there is an overall lack of demonstrated intra- and inter-laboratory reproducibility (criterion 4). It should be considered that the overall scoring of these three criteria reflects the innovative nature of the research field, in which providing sufficient details for others to reproduce findings or further develop a platform is essential, while the broader implementation of presented models, for example by pharmaceutical companies or contract research organizations (hence inter-laboratory reproducibility), does not necessarily result in additional publications.

Comparing the top three high- versus low-scoring studies, the greatest discrepancies are observed in criteria 6-9, where the top three studies have a 4.5- to 10-fold higher score than the low-scoring studies. The largest discrepancy is found for the criterion ‘Have assays/analyses to determine a relevant function been performed?’ (1.67 versus 0.17), which may relate to two other low-scoring criteria that relate to disease modeling and *in vitro*/*in vivo* extrapolation. Thus, the translational aspects of the study (function and *in vivo–in vitro*) appear to be important for overall study quality. Future studies combining *in vitro* and *in silico* modeling, like the virtual twin approach, may bridge this translational gap and extend the approach for various translational pharmacology applications, such as toxicity assessment, first-in-human dosing and metabolism studies.

Additionally, insufficient description of materials for chip generation is a low-scoring criterion. Although such information is essential for reproducibility, it should be noted that, as discussed earlier, for commercial OoC platforms, such detailed information is not always made available.

### Strengths and limitations

We have performed an extensive systematic review with rigorous methodology, with *a priori* protocol registration, screening, data extraction and quality appraisal by two reviewers. Our extensive search strategy was performed in the two main biomedical databases, leading to a large number of references, validated by cross-checking in other articles. To our knowledge, this is the first systematic review exploring all evidence available on KoC models. Owing to heterogeneity across included studies regarding used cell types, represented nephron segments, microfluidic platforms and functional analyses, meta-analyses could not be performed. Our extensive quality/risk of bias analysis suggests that there are common shortcomings across the included studies. Although these shortcomings are partially intrinsically related to this rapid-developing and pioneering field of research, we recommend that researchers more specifically address the intra- and inter-laboratory reproducibility, and translational capacity of developed models.

## Conclusions

Overall, KoC models have evolved from relatively straightforward models using a single cell type in in-house developed flow platforms to more complex models combining multiple human organ models mimicking systemic (patho-)physiological processes. The availability of commercial chip platforms and human (induced)stem cells and organoids have contributed to the establishment of a broad range of kidney-based OoC and MOC models to study disease mechanisms and assess drug efficacy and toxicity, even in a personalized medicine setting. Studies highlighting the predictive power of OoC models over conventional (animal) models in the assessment of drug-related clinical toxicity and efficacy testing, allowing intra- and inter-laboratory reproducibility and translational opportunities, are needed. With these challenges ahead, the described developments offer great promise towards the Reduction, Refinement and Replacement of animal models in kidney and drug research.

## MATERIALS AND METHODS

The protocol of our systematic review was registered *a priori* in PROSPERO (CRD42022323103).

### Study selection

We systematically searched with Medical Subject Headings (MeSH) terms and free-text terms in MEDLINE and Embase (Supplementary Materials and Methods). All *in vitro*, *in silico* or material-based studies reporting mimicking (1) an aspect of human renal function or physiology, or (2) combining such models of (parts of) the kidney with models for other organs or tissues, were included. Published articles from the same author group, with similar experimental design, resulting in different outcomes were included as separate article. Articles employing a chip platform without cells and/or dynamic flow were excluded. Non-English publications, conference abstracts and editorials were excluded. Two researchers independently evaluated the eligibility of retrieved studies, based on title and abstract. Next, selected studies were reviewed in the full-text screening phase. Discrepancies between reviewers were resolved by discussion and, if needed, by adjudication by a third reviewer.

### Data extraction and quality assessment

Data on study characteristics, including cell type, kidney segment, viability, function, chip design and organ combinations, were extracted by two researchers. As no specific quality instrument for organ-on-a-chip exists, we performed the quality assessment based on criteria adapted from Irvine et al. (2021), including (1) cell/tissue/organoid source; (2) functional parameters; and (3) platforms. Based on ten criteria (Table S3), scored 0, 1, or 2 points when not, partially or fully meeting the criterion, respectively, the score provides an indication of the quality of reporting and potential reproducibility and external validation. Averages of the scores from two of the authors were used for quality assessment.

### Data synthesis

We have summarized data across the included studies descriptively, and performed subgroup analyses based on platform characteristics such as represented nephron segments, cell source and type, number of organs combined and functional measurements. A sensitivity analysis including solely studies with low risk of bias was conducted. We decided not to perform a meta-analysis because no common outcome and excessive heterogeneity were identified across the included studies.

## Supplementary Material

10.1242/dmm.050113_sup1 Supplementary information
