# Supplementary material for: A systematic review of kidney-on-a-chip-based models to study human renal (patho-)physiology
Source: Dis Model Mech. 2023 Jun 19;16(6):dmm050113. doi: 10.1242/dmm.050113 (PMC10309579; doi:10.1242/dmm.050113)
Supplement: Supplementary information [file dmm-16-050113-s1.pdf]

Table S1. Excluded studies

Click here to download Table S1

Table S2. Included studies and analysis criteria

Click here to download Table S2

Table S3. Questions for quality assessment and assessment guide

Click here to download Table S3

Table S4. Quality assessment of included studies

Click here to download Table S4

Supplementary Materials and Methods

Literature search

Search strategy on PubMed (performed on 22-11-2022)#	Search	# ref
#1	("Kidney Diseases"[Mesh]) OR "Renal Replacement Therapy"[Mesh]) OR "Renal Insufficiency"[Mesh]) OR "Renal Insufficiency, Chronic"[Mesh])	687,740
#2	(dialysis[tiab]) OR hemodialysis[tiab] OR haemodialysis[tiab] OR hemofiltration[tiab] or haemofiltration[tiab] OR hemodiafiltration[tiab] or haemodiafiltration[tiab] OR dialysate*[tiab]) OR (kidney*[tiab] OR renal*[tiab] OR nephron*[tiab] OR glomerul*[tiab] OR ESRF[tiab] OR ESKF[tiab] OR ESRD[tiab] OR ESKD[tiab] OR CKF[tiab] OR CKD[tiab] OR CRF[tiab] OR CRD[tiab] OR CAPD[tiab] OR CCPD[tiab] OR APD[tiab] OR Tubul*[tiab] OR Henle*[tiab] OR tubuloid[tiab]	1,247,089
#3	#1 OR #2	1,396,164
#4	Chip*[tiab] OR multi-organ-on-a-chip[tiab] OR kidney-on-a-chip[tiab] OR organ-on-a-chip[tiab] OR renal-on-a-chip[tiab] OR organoid-on-a-chip[tiab] OR glomerulus-on-a-chip[tiab] OR microdevice[tiab] OR microphysiologic*[tiab] OR Microfluid*[tiab] OR microsystem*[tiab] OR chip-based[tiab] OR biomimetic[tiab] OR microengine*[tiab]	124,396
#5	"Lab-On-A-Chip Devices"[Mesh] OR "Cells, Immobilized"[Mesh]	9,974
#6	#4 OR #5	128,030
#7	#3 AND #6	**2,692**

Search strategy on Embase (performed on 22-11-2022)

#	Search	# ref
#1	'kidney disease'/exp OR 'renal replacement therapy'/exp OR 'kidney failure'/exp OR 'chronic kidney failure'/exp	1,282,493
#2	dialysis:ti,ab OR hemodialysis:ti,ab OR haemodialysis:ti,ab OR hemofiltration:ti,ab or haemofiltration:ti,ab OR hemodiafiltration:ti,ab or haemodiafiltration:ti,ab OR dialysate*:ti,ab OR kidney*:ti,ab OR renal*:ti,ab OR nephron*:ti,ab OR glomerul*:ti,ab OR ESRF:ti,ab OR ESKF:ti,ab OR ESRD:ti,ab OR ESKD:ti,ab OR CKF:ti,ab OR CKD:ti,ab OR CRF:ti,ab OR CRD:ti,ab OR CAPD:ti,ab OR CCPD:ti,ab OR APD:ti,ab OR Tubul*:ti,ab OR Henle*:ti,ab OR tubuloid:ti,ab	1,713,997
#3	#1 OR #2	2,096,352
#4	Chip*:ti,ab OR multi-organ-on-a-chip:ti,ab OR kidney-on-a-chip:ti,ab OR organ-on-a-chip:ti,ab OR renal-on-a-chip:ti,ab OR organoid-on-a-chip:ti,ab OR glomerulus-on-a-chip:ti,ab OR microdevice:ti,ab OR microphysiologic*:ti,ab OR Microfluid*:ti,ab OR microsystem*:ti,ab OR chip-based:ti,ab OR biomimetic:ti,ab OR microengine*:ti,ab	141,516
#5	'lab on a chip'/exp OR 'immobilized cell'/exp	14,534
#6	#4 OR #5	148,332
#7	#3 AND #6	**4,276**
